# Results of a national UK physician reported survey of COVID-19 infection in patients with a myeloproliferative neoplasm

**DOI:** 10.1038/s41375-021-01143-2

**Published:** 2021-02-12

**Authors:** Richard A. Salisbury, Natalia Curto-Garcia, Jennifer O’Sullivan, Frederick Chen, Paolo Polzella, Anna L. Godfrey, James Russell, Steven Knapper, John Willan, Rebecca Frewin, Shivani Joshi, Siamak Arami, Sarah Burns, Chun Huat Teh, Frances Wadelin, Jaymathi Dhanapal, Pratap Neelakantan, Dragana Milojkovic, Beth Psaila, Richard Szydlo, Sebastian Francis, Catherine Cargo, Manish Jain, Andrew McGregor, Louise Wallis, Andrew Duncombe, Hayder Hussein, Peter Dyer, Laura Munro, Lee Bond, Mary Frances McMullin, Tim C. P. Somervaille, Mamta Garg, Mallika Sekhar, Claire Harrison, Adam J. Mead, Andrew J. Innes

**Affiliations:** 1 NIHR Biomedical Research Centre and MRC Molecular Haematology Unit, Weatherall Institute of Molecular Medicine, University of Oxford, Oxford, UK; 2 Department of Haematology, Guys and St Thomas’ NHS Foundation Trust, London, UK; 3 Department of Haematology, Barts Health NHS Trust, London, UK; 4 Department of Haematology, Buckinghamshire Healthcare NHS Trust, Aylesbury, UK; 5 Department of Haematology, Cambridge University Hospitals NHS Foundation Trust, Cambridge, UK; 6 Division of Cancer and Genetics, Cardiff University, Cardiff, UK; 7 Department of Haematology, Frimley Health NHS Foundation Trust, Slough, UK; 8 Department of Haematology, Gloucestershire Hospitals NHS Foundation Trust, Gloucester, UK; 9 Department of Haematology, London Northwest Healthcare University NHS Trust, Harrow, UK; 10 Department of Haematology, Manchester University NHS Foundation Trust, Manchester, UK; 11 Department of Haematology, Western General Hospital, Edinburgh, UK; 12 Department of Haematology, Nottingham University Hospitals NHS Trust, Nottingham, UK; 13 Department of Haematology, Royal Berkshire NHS Foundation Trust, Reading, UK; 14 Centre for Haematology, Imperial College London, London, UK; 15 Department of Haematology, Sheffield Teaching Hospitals NHS Foundation Trust, Sheffield, UK; 16 Department of Haematology, The Leeds Teaching Hospitals NHS Trust, Leeds, UK; 17 Department of Haematology, The Newcastle upon Tyne Teaching Hospitals NHS Foundation Trust, Freeman Hospital, Newcastle, UK; 18 Department of Haematology, The Royal Bournemouth and Christchurch Hospitals NHS Foundation Trust, Bournemouth, UK; 19 Department of Haematology, University Hospital Southampton NHS Foundation Trust, Southampton, UK; 20 Department of Haematology, University Hospitals Birmingham NHS Foundation Trust, Birmingham, UK; 21 Department of Haematology, University Hospitals of North Midlands NHS Trust, Stoke, UK; 22 Department of Haematology, York Teaching Hospital NHS Foundation Trust, York, UK; 23 Centre for Medical Education, Queen’s University, Belfast, UK; 24 Leukaemia Biology Laboratory, Cancer Research UK Manchester Institute, The University of Manchester, Manchester, UK; 25 The Christie NHS Foundation Trust, Manchester, UK; 26 Department of Haematology, University Hospitals of Leicester NHS Foundation Trust, Leicester, UK; 27 Department of Haematology, Royal Free London NHS Foundation Trust, London, UK; 28 Department of Haematology, University College London Hospitals NHS Foundation Trust, London, UK

**Keywords:** Epidemiology, Risk factors, Disease-free survival, Myeloproliferative disease, Myeloproliferative disease

The COVID-19 pandemic caused by the spread of SARS-CoV-2 virus has already had a catastrophic impact with more than 1.4 million deaths worldwide as of 29 November 2020 [1]. Many countries including the UK attempted to control spread of the virus through nationwide lockdowns. In the UK, “shielding” was implemented to protect those deemed to be in high-risk groups including those over 75, and those with haematological malignancies, including myeloproliferative neoplasms (MPN). Haematological malignancies have been associated with increased risk of COVID-19-related death [2]. However, these disorders are heterogeneous, and shielding is challenging for patients to maintain for prolonged periods, particularly when dealing with a poorly understood risk. While MPN patients are known to have an increased risk of requiring hospital admission with infections [3], the heterogeneity of this population, combined with the limited knowledge of SARS-CoV-2, means there is likely to be a spectrum of risk of death from COVID-19 in MPN patients. With the imminent threat of further waves of infection, or chronic low-level population transmission, experience-based risk quantification to inform clinical practice and patient information is needed.

In order to provide further data on the consequences of COVID-19 infection in patients with MPN, we conducted a national survey coordinated by members of the National Cancer Research Institute (NCRI) MPN subcommittee. Members of the NCRI MPN subgroup coordinated data collection on patients from their own centre and local hospitals. Clinicians working in areas of the UK not well represented by the MPN subgroup membership were also contacted directly. Outcome data on thrombosis, bleeding, and mortality were collected as well as information on baseline characteristics, MPN treatment and infection severity. The case report form, shown in Supplementary Table 1, was distributed to centres and physicians had discretion to select the most appropriate options from dropdown menus. Data were fully anonymised locally and collated within a central database. Analysis was conducted using SPSS (IBM Corp., Armonk, NY). Categorical data were compared using two-sided *Χ*^2^ or Fisher’s exact test and continuous non-parametric data with Kruskall–Wallis test. Survival analysis was performed using Kaplan–Meier estimation with group comparison by log rank test for univariable analysis and Cox proportional-hazard regression was used for multivariable analysis. Age standardised mortality rate was calculated using the 2013 European Standard Population as a reference. Age standardised mortality = ${\sum} {\left( {P_km_k} \right)} /{\sum} {P_k}$ where *P*_*k*_ = standard population in group *k* and *m*_*k*_ = age-specific mortality rate in group *k*.

Haematologists at 42 hospitals were contacted. Replies were received from 30 hospitals with 27 centres reporting one or more cases. In total, data were received on 77 patients with known MPN (essential thrombocythemia (ET), *n* = 28, polycythemia vera (PV), *n* = 18, primary or secondary myelofibrosis (MF), *n* = 27, and MPN unclassifiable or MDS/MPN overlap syndrome, *n* = 4) with a diagnosis of COVID-19 infection prior to 5 July 2020.

Patient demographics are presented in Table 1. Median age was 74 years (IQR 63.5–82 years) and 45 (58%) patients were male. Men were over-represented in our cohort, potentially reflecting an increased susceptibility to infection or severe infection (leading to reporting bias) with COVID-19, especially for those with ET where male preponderance is not expected. The ISARIC study is a large, prospective observational study of patients hospitalised with COVID-19 [4]. Median age was 73 years and 57% were male, similar to our MPN cohort, therefore as age and male sex are known predictors of outcome from COVID-19, ISARIC has been included in Table 1 for comparison [5]. ISARIC is not a perfect comparator however and contains a smaller proportion of patients with hypertension and diabetes than our MPN cohort, which are also recognised predictors of outcome from COVID-19 infection.

**Table 1:** Patient characteristics and outcomes for subgroups of MPN and for hospitalised patients.

		ET	PV	MF	Other	*p*^a^	Total	Outpatient	Inpatient	ISARIC^b^	*p*^c^
Number	*n*	28(36%)	18(23%)	27(35%)	4(5%)		77	14(18%)	63(82%)	60,430	
Age	Median (IQR)	78(71–84)	73(54–76)	73(61–81)	81(57–88)	0.06	74(64–82)	63.5(51–79)	75(67–82)	73	
Gender	Female	12(43%)	7(39%)	13(48%)	0	0.38	32(42%)	7(50%)	25(40%)	25,899(43%)	0.6
	Male	16(57%)	11(61%)	14(52%)	4(100%)		45(58%)	7(50%)	38(60%)	34,422(57%)	
Ethnicity	Asian	2(7%)	2(11%)	1(4%)	0	0.96	5(7%)	1(7%)	4(6%)		
	Black	1(4%)	1(6%)	1(4%)	0		3(4%)	1(7%)	2(3%)		
	White	24(89%)	15(83%)	25(93%)	4(100%)		68(89%)	12(86%)	56(90%)		
Active treatment	Observation only	2(7%)	1(6%)	3(11%)	1(25%)	0.53	7(9%)	2(14%)	5(8%)		
	Active treatment	26(93%)	17(94%)	24(89%)	3(75%)		70(91%)	12(86%)	58(92%)		
	Ruxolitinib	2(7%)	4(22%)	19(70%)	1(25%)	**<0.01**	26(34%)	3(21%)	23(37%)		
Number of additional co-morbidities	None	9(32%)	8(44%)	12(44%)	1(25%)	0.14	30(39%)	9(64%)	18(29%)		
	1 or 2	9(32%)	9(50%)	13(48%)	3(75%)		34(44%)	4(29%)	30(48%)		
	3 or 4	10(36%)	1(6%)	2(7%)	0		13(17%)	1(7%)	15(24%)		
Co-morbidity	Hypertension	14(50%)	4(22%)	12(44%)	1(25%)	0.25	31(40%)	3(21%)	28(44%)	16,074(32%)	**0.03**
	Diabetes	8(29%)	3(17%)	5(19%)	0	0.63	16(21%)	1(7%)	15(24%)	9617(14%)	**0.02**
Hospital admission	Outpatient	1(4%)	6(33%)	6(22%)	1(25%)	**0.04**	14(18%)				
	Inpatient	27(96%)	12(67%)	21(78%)	3(75%)		63(82%)				
ICU admission	Not known	0	0	1(4%)	1(25%)	0.79	1(1%)		2(3%)		
	Not indicated	23(82%)	15(83%)	23(85%)	3(75%)		63(82%)		50(79%)		
	Admitted to ICU	5(18%)	3(17%)	3(11%)	0		11(14%)		11(18%)	9754(16%)	0.73
Maximal respiratory support	None	3(11%)	9(50%)	9(33%)	2(25%)	0.14	23(30%)	14(100%)	9(14%)		
	Oxygen	15(54%)	6(33%)	11(41%)	2(50%)		34(44%)	0	34(54%)		
	High Flow	5(18%)	0	2(7.4%)	0		7(9%)	0	7(11%)		
	CPAP/NIV	1(4%)	0	3(11%)	0		4(5%)	0	4(6%)		
	Intubation	4(14%)	3(17%)	2(7%)	0		9(12%)	0	9(14%)	5643(10%)	0.29
Mortality	Died	13(46%)	8(44%)	12(44%)	2(50%)	1	35(45%)	2(14%)	33(52%)	17,031(28%)	**<0.01**
	Ongoing	1(4%)	0	1(4%)	0		2(3%)	1(7%)	1(2%)	3209(5%)	0.26
	Recovered	14(50%)	10(56%)	14(52%)	2(50%)		40(52%)	11(79%)	29(46%)	26,191(57%)	0.09
Other outcomes	Venous thrombosis	1(PE; 4%)	0	1(PE; 4%)	0	0.98	2(3%)	0	2(3%)		
	Arterial thrombosis	1(CVA; 4%)	1(MI; 6%)	0	0	0.66	2(3%)	0	2(3%)		
	Bleed	2(MB + NMB; 7%)	0	1(NMB; 4%)	0	1	3(4%)	0	3(5%)		

*p* values < 0.05 are highlighted in bold.

*CVA* cerebrovascular accident, *ET* essential thrombocythemia, *IQR* interquartile range, *MB* major bleed (>2 units RBC or intracerebral), *MI* myocardial infarction, *MF* myelofibrosis (primary and secondary), *NMB* non-major bleed (required medical intervention/<3 units RBC transfusion), *PE* pulmonary embolism, *PV* polycythemia rubra vera.

^a^Fisher’s exact test (two-sided) or Kruskall–Wallis test comparing MPN subgroups.

^b^Most recent results of ISARIC study [6].

^c^*Χ*^2^ test (two-sided) comparing inpatient and ISARIC cohorts.

BMI was available in 45 (56%) patients and mean BMI was 24.5 (95% CI 23.3–25.7). In addition to MPN, most patients had at least one other significant co-morbidity (median number was 1 (IQR 0–2)), while 20 (26%) patients had no additional co-morbidities. The most frequent co-morbidities were hypertension (*n* = 31, 40%), diabetes (*n* = 16, 21%), and cerebrovascular disease (*n* = 15, 19%). No statistically significant differences were found in age, sex, ethnicity, or co-morbidity between MPN subtypes.

The vast majority of patients were receiving active treatment for their MPN with only seven (9%) patients under watchful waiting. Patients not receiving cytoreductive therapy are underrepresented in this cohort [6], possibly reflecting a lower risk of developing severe COVID-19 infection, but this requires further investigation. Twenty-six (34%) patients were on ruxolitinib, 38 (49%) on hydroxycarbamide, 4 (5%) on anagrelide, and 2 (3%) on interferon. The indication for ruxolitinib was MF in 19 (73%) of 26 patients. Ruxolitinib treated MPN patients may be over-represented in this cohort [7], therefore these patients could have increased risk of developing symptomatic COVID-19 infection, although this remains speculative and requires further exploration in independent studies. Antiplatelet agents were being taken by 46 (60%) patients with 15 (20%) on full anticoagulation.

Infections were diagnosed between 11 March 2020 and 12 June 2020. Symptoms started a median of 4 days before diagnosis (IQR 2–7 days) and were similar to previous reports: 55 (71%) presenting with dyspnoea, 51 (66%) cough, 46 (60%) fever, 10 (13%) diarrhoea, 9 (12%) myalgia, and 3 (4%) nausea and vomiting.

COVID-19 infection was predominantly contracted in the community (*n* = 55, 71%), but 22 (29%) had physician reported hospital acquired infections. Infections peaked on 4 April 2020 in our cohort, similar to the UK peak on 5 April 2020 [8]. Hospital admission was required by 63 (82%) patients. Diagnosis was made by viral PCR test in 59 (77%) patients, imaging only in 8 (10%) and 6 (8%) were diagnosed clinically (missing data in 4). WHO severity score at presentation was Mild, Moderate, Severe or Critical in 20 (26%), 19 (25%), 21 (27%), and 12 (16%) respectively. Five patients had missing data. Of the 11 (14%) patients requiring ICU admission, two (18%) received non-invasive ventilation, nine (82%) intubation, and four (36%) renal replacement therapy. Antibiotics were given to 54 (70%) patients but only three (4%) received azithromycin as a treatment for COVID-19. No COVID-19 experimental treatments or repurposed established treatments such as dexamethasone were given. Treatment for MPN was continued in 39 (51%) patients and 32 (42%) stopped MPN therapy, of whom nine stopped ruxolitinib.

Thrombosis occurred in four (5%) patients, two had a pulmonary embolism and two an arterial thrombosis. Neither of the patients who developed a PE had a history of venous thromboembolism (VTE) and were not taking therapeutic anticoagulation (one was taking aspirin) prior to admission. Both required enhanced respiratory support, one CPAP and the other intubation during admission. One patient developed a myocardial infarction whilst taking a direct oral anticoagulant for atrial fibrillation and one an ischaemic stroke whilst intubated in intensive care. Bleeding occurred in three (4%) patients. All patients who had a bleeding event were taking aspirin and two patients had thrombocytopenia. Both patients with thrombocytopenia were receiving cytoreductive treatment for ET.

At the time of data analysis 35 of 77 (45%) patients had died, 40 (52%) had recovered, and 2 had ongoing illness. The median follow-up for survivors was 74.5 days, and no deaths occurred after 32 days. The overall survival for the group at 32 days was 54% (Fig. 1A). Survival was significantly different between those admitted to hospital and those remaining in the community (45 vs 86% *p* = 0.02, Fig. S1a). Age standardised mortality rate for MPN patients who required hospitalisation with COVID-19 infection was 18,716 per 100,000 population (95% CI 12,330–25,102 per 100,000) and 14,058 deaths per 100,000 for the ISARIC cohort (95% CI 13,846–14,271 per 100,000). COVID-19 was the primary cause of death in 33 (94%) and a contributing factor in 1 (3%). One death was due to transformation to AML. Age over 65 years was associated with a significantly worse overall survival (44 vs 80%, *p* = 0.005, Fig. 1B), and while numbers are low, there were no deaths in anyone under 50 (*n* = 5). COVID-19 disease severity at presentation was also associated with a worse outcome (survival by group, mild 100%, moderate 63%, severe 29%, and critical 25%, *p* < 0.001, Fig. 1C). Unexpectedly, there was no difference in survival by MPN type (54%, 56%, 56%, and 50% for ET, PV, MF, or other MPN, respectively, *p* = 0.983, Fig. 1D). The effect of age remained an important factor in all disease groups (Fig. 1E). The comparable survival between MPN subgroups is surprising, as is the high proportion of ET patients that required inpatient care (96%). Although it is possible that this is reflective of an increased susceptibility to COVID-19 infection in ET patients, as recently reported by Wang et al. [9], this may equally be accounted for by an uneven age distribution between MPN subtypes and small sample size. After adjustment for age, there was a non-significant trend for ET patients to have a superior outcome (Fig. S1b), which becomes more evident when COVID-19 severity is accounted for (Fig. S1c).

**Fig. 1:**
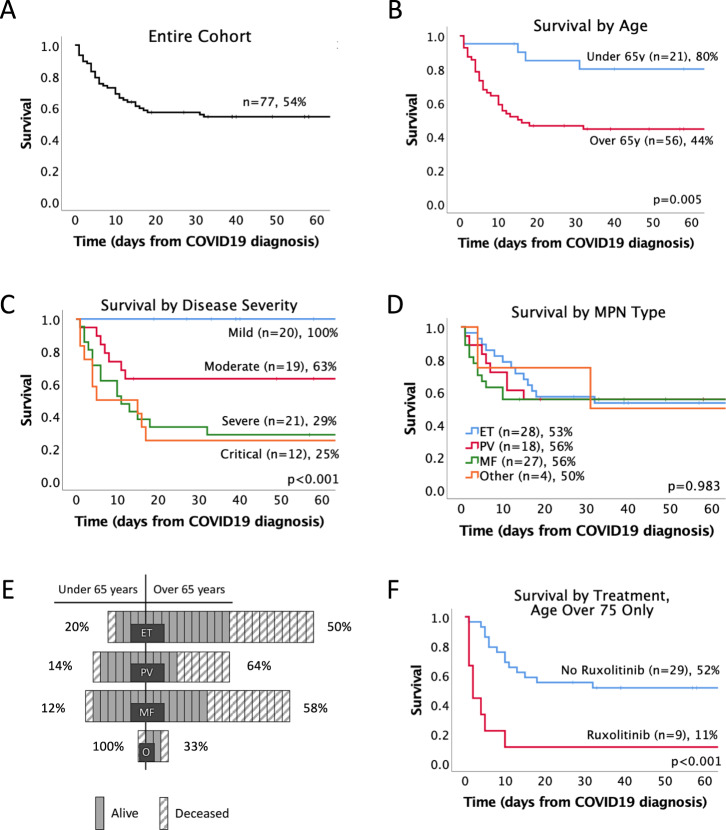
Survival outcome following symptomatic COVID-19 infection in patients with MPN. Kaplan–Meier estimate of survival for entire MPN patient cohort covering time from COVID-19 diagnosis censored at date of data submission (**A**). Effect of age (**B**), COVID-19 severity on admission according to WHO criteria (**C**), and MPN subtype (**D**). Effect of age by MPN subtype, each box represents a patient, solid fill box denotes survival and hatched box death (**E**). Effect of ruxolitinib treatment in patients aged over 75 years (**F**). ET essential thrombocythemia, PV polycythemia vera, MF myelofibrosis (including primary and secondary), O other MPN (including MDS/MPN overlap and MPN unclassifiable).

There was no impact on survival by antiplatelet, anticoagulant (data not shown), or hydroxycarbamide use (55 vs 53%, *p* = 0.569, Fig. S1d). However, there was an age dependent effect of ruxolitinib therapy on survival, with no significant impact in those under 75 years (64 vs 68%, *p* = 0.727, Fig. S1e), but a significantly poorer outcome in those over 75 years (11 vs 52%, *p* < 0.001, Fig. 1f). Patients taking ruxolitinib at the onset of COVID-19 infection were included in this analysis, including those that subsequently stopped. There was no survival impact from discontinuing ruxolitinib, but numbers were small.

Although observational data such as the current cohort might be associated with certain biases, including the potential selective reporting of more severe cases, we believe that a number of important conclusions can be drawn from these data. First, MPN patients admitted with COVID-19 infection appear to have worse outcomes than expected when compared to the ISARIC study, although there are significant differences in baseline co-morbidities (Table 1). Second, age is highly predictive of outcome. Young MPN patients, particularly those undergoing observation only, were underrepresented in the cohort suggesting that they either have milder infection or are less susceptible to infection than older patients with MPN. Therefore, younger MPN patients might not be at increased risk compared to the general population, although it is important to note that numbers included are small. However, older MPN patients may be at higher risk of death than the age-matched population and therefore are more likely to benefit from strict social distancing or shielding. Furthermore, comparison with a recently published large cohort of chronic lymphocytic leukemia (CLL) shows similar outcomes for MPN and CLL patients [10]. The outcome of MPN patients in this study is worse than an Italian cohort of haematological malignancy [11], where hospitalised MPN patients with COVID-19 had a mortality rate of 33 vs 46% in the current cohort. The reasons for this difference are unclear although little granularity is available from the Italian cohort for further comparison.

Data on thrombotic risk from COVID-19 infection are highly variable, however a series of hospitalised patients with a greater proportion of patients admitted to ICU found rates of VTE at 5% and arterial thrombosis at 3% [12], similar to our MPN cohort. This suggests that MPN patients may not have an increased rate of thrombotic complications, possibly due to the high proportion of patients on antiplatelets or anticoagulation, but larger prospective studies are required to confirm this.

The poor outcome of elderly patients pre-treated with ruxolitinib is striking and ruxolitinib treated patients account for one-third of the cohort, including 70% of MF patients, more than would be expected based on real-world UK data [7]. Although ruxolitinib is immunosuppressive, it also has potential beneficial anti-inflammatory effects [13] and has recently been proposed as a therapy for COVID-19 associated hyperinflammation and respiratory distress. It is not possible to determine whether poor outcome in ruxolitinib treated patients in our study is a direct result of the drug, or whether ruxolitinib therapy is a surrogate of severe MPN.

In summary, our data support that older MPN patients, particularly those receiving ruxolitinib, might be at increased risk of adverse outcomes following COVID-19 infection. Younger patients under observation may not be at increased risk in comparison with the general population and stringent shielding measures may not be required for this patient group. It remains unknown how many patients with MPN developed COVID-19 infection and it is very likely many infections occurred in the community and were not diagnosed, as early in the outbreak testing was mostly limited to inpatients in the UK. Serology surveys of MPN patients will be informative in this regard. Our study provides useful data to inform evidence-based risk stratification of MPN patients with regards to risk of COVID-19 infection.

## Supplementary information

Supplemental figure 1

Supplemental table 1
