# Supplementary material for: Results of a national UK physician reported survey of COVID-19 infection in patients with a myeloproliferative neoplasm
Source: Leukemia. 2021 Feb 12;35(8):2424–30. doi: 10.1038/s41375-021-01143-2 (PMC7880652; doi:10.1038/s41375-021-01143-2)

Supplementary Figure 1 – Survival stratified by inpatient or outpatient treatment (a), Cox-proportional hazard analysis of survival adjusted for age (b), and additionally WHO severity (c). Analysis of MPN treatment on survival; hydroxycarbamide (d), and ruxolitinib in patients aged less than 75 years (e). Abbreviations: ET=Essential Thrombocythemia; HC=Hydroxycarbamide; HR=Hazard Ratio; PV=Polycythemia Vera; MF=Myelofibrosis (Primary and Secondary).

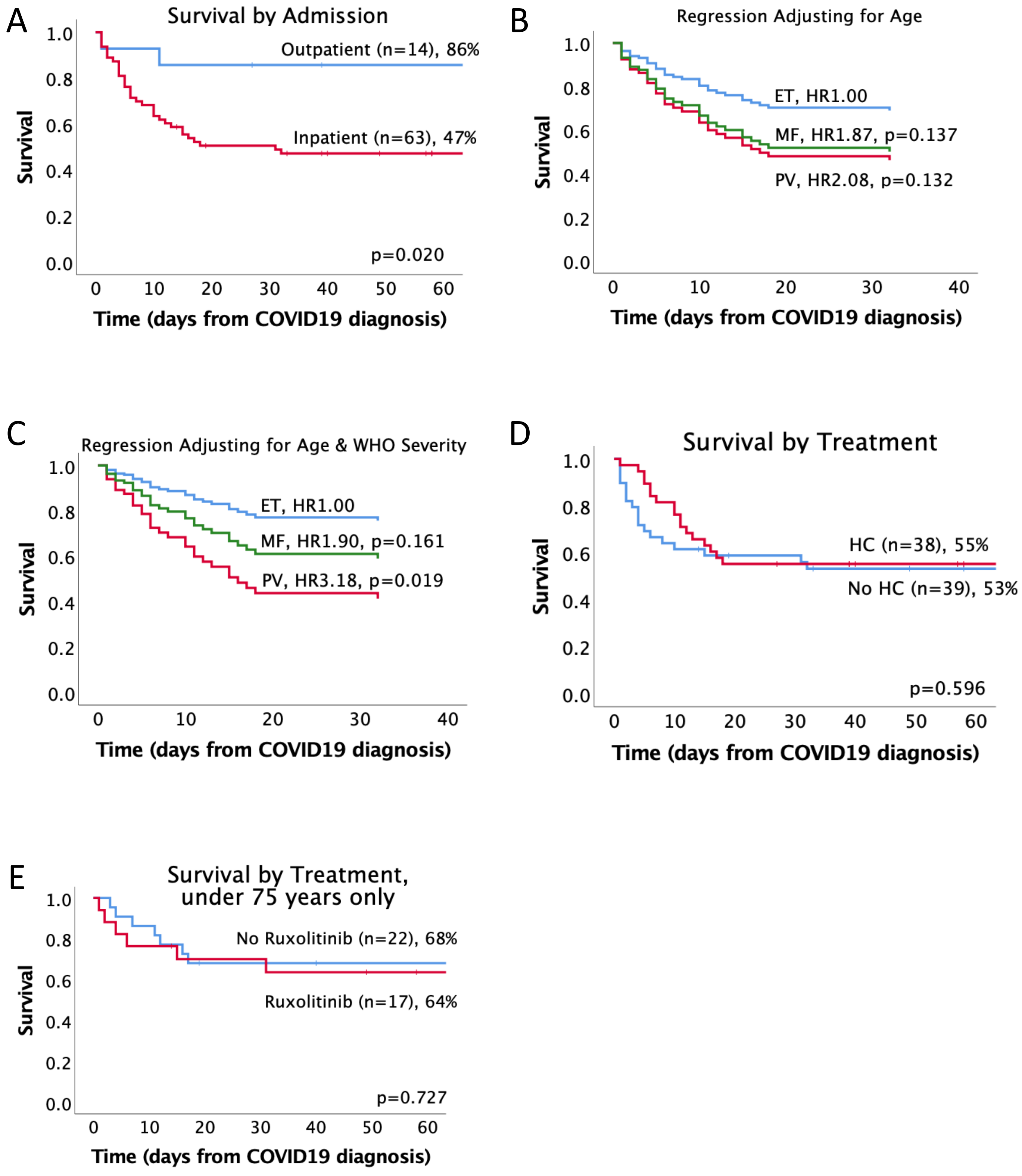

Supplement: Supplementary file 1 — Supplemental figure 1 [file 41375_2021_1143_MOESM1_ESM.pdf]
